# Microbial Decontamination of Beef Carcass Surfaces by Lactic Acid, Acetic Acid, and Trisodium Phosphate Sprays

**DOI:** 10.1155/2020/2324358

**Published:** 2020-11-02

**Authors:** Khalid Ibrahim Sallam, Samir Mohammed Abd-Elghany, Mohammed Abdullah Hussein, Kálmán Imre, Adriana Morar, Alaa Eldin Morshdy, Mohamed Zakaria Sayed-Ahmed

**Affiliations:** ^1^Department of Food Hygiene and Control, Faculty of Veterinary Medicine, Mansoura University, Mansoura, Egypt; ^2^Food Control Department, Faculty of Veterinary Medicine, Zagazig University, Zagazig, Egypt; ^3^Department of Animal Production and Veterinary Public Health, Faculty of Veterinary Medicine, Banat's University of Agricultural Sciences and Veterinary Medicine “King Michael I of Romania” Timişoara, 300645, Romania; ^4^Department of Internal Medicine and Infectious Diseases, Faculty of Veterinary Medicine, Mansoura University, Mansoura, Egypt; ^5^Department of Clinical Pharmacy, College of Pharmacy, Jazan University, Jizan, Saudi Arabia

## Abstract

The present study was undertaken to investigate the effect of lactic acid (LA), acetic acid, (AA) and trisodium phosphate (TSP) spray on the microbiological population of beef carcass surfaces slaughtered in a traditional abattoir in Zagazig, Egypt. Higher microbial populations were determined on the shoulder than on the thigh surfaces, and meat sampling by tissue excision technique yielded significantly higher (*P* < 0.01) microbial count than swabbing method. The application of LA (2%), AA (2%), and TSP (12%) sprays for 30 seconds significantly (*P* < 0.01) reduced the microbial population counts on the beef surfaces by 0.9 to 2.2 logs. A complete inhibition of enterococci growth was achieved by LA and AA sprays. In general, LA and AA sprays were more efficient as antimicrobial agents than the TSP spray. Among the studied organisms, enterococci were the most reducible bacteria by LA and AA, followed by *Enterobacteriaceae* and coliforms, while *Staphylococcus aureus* being the least. This study also indicated that microbial populations determined on the shoulder were higher than on the thigh surfaces, and meat sampling by tissue excision technique yielded significantly higher (*P* < 0.01) microbial count than swabbing method.

## 1. Introduction

Contamination of meat carcasses during slaughtering and processing is an inevitable process. Beef carcasses can be easily contaminated with various pathogenic bacteria providing from the surrounding environment. In this regard, within several slaughtering procedures, including dressing, evisceration, processing, and storage, the sources of the microbial contamination can be considered: the skin, feet, stomach, intestinal contents, hands, clothes of employees, equipment, and water used for washing carcasses, as well as dust and air [1–4].

The extent to which beef carcasses are contaminated with bacteria is influenced mostly by variation among processing plants, including their design, slaughtering speed, and the operator's skills. The occurred contamination level reflects the extent to which hygiene and sanitation standards are applied during slaughtering, while the composition of the flora reflects the various sources of contamination and the effectiveness of the applied hygienic measures [5–7].

Although many modern abattoirs equipped with modern and sophisticated machinery and equipment are in service in Cairo and some other big cities in Egypt, slaughtering in the majority of the Governorates, including Sharkia (Zagazig), is still applied in the old traditional abattoirs. In this slaughtering units, the dressing of cattle carcasses is conducted on the floor, along with the poorly applied hygienic standards that can lead to a greater microbial contamination of the meat, which mostly retailed in the domestic market, where is stored at abusive temperature to be sold directly to the consumers [8].

In the case of the existence of extensive contamination or abusive conditions that allow bacteria to reproduce usually increases the risk for the presence of pathogenic bacteria and formation of toxins in a food matrix. In response to that situation, regulatory authorities, researchers, and food safety managers in beef industry addressed food safety concerns by developing a variety of methods that are now implemented or are being further developed, in order to improve the microbiological quality [2, 4, 9–11]. In this regard, as has been suggested by several authors [9, 11, 12], the using of chemical decontaminants can be a promising tool.

Accepted decontamination solutions should not have adverse effects on food workers' health during their application or on consumers, as a result of their use [12]. Decontamination systems, based on the using of chemical agents, are approved by the USDA Food Safety and Inspection Service, as a component of the implemented HACCP plan, if the chemicals are (i) “Generally Recognized as Safe” (GRAS) by the Food and Drug Administration, (ii) do not create an “adulterant” situation, (iii) do not create labeling issues (i.e., “added ingredients”), and (iv) can be supported by scientific studies as being effective [13].

The most frequently used chemical decontaminants (e.g., acetic and lactic acids) are solutions of organic acids (1-3%), which reduce numbers of bacteria on carcass tissue [14, 15]. In addition to organic acids, several other chemical solutions (e.g., chlorine and chlorine dioxide, trisodium phosphate, hydrogen peroxide, ozone, sodium bisulfate, sodium chloride, nisin, and potassium sorbate) have been proposed and tested (some have been approved) for their using in decontamination systems [16, 17].

The present study was undertaken to investigate the effect of lactic acid (LA), acetic acid (AA), and trisodium phosphate (TSP), as safe organic decontaminating agents, on the microbiological population of aerobic bacteria, including *Enterobacteriaceae*, enterococci, coliforms, and *Staphylococcus aureus* present on the beef carcasses, obtained in an old traditional abattoir in Zagazig, Egypt. In order to fulfill the main objectives of the present study, a preliminary investigation was done that is aimed at defining the better strategy between tissue excisions and swabbing techniques to determine the microbial counts on beef carcass surfaces under the influence of the chemical decontaminants.

## 2. Materials and Methods

### 2.1. Carcass Preparation and Chemical Treatments

Beef carcasses were obtained after a slaughtering process, according to the Islamic method at the Zagazig traditional abattoir, Egypt. The implementation of hygienic standards is lacking in such abattoirs, in which the live animals and dressed carcasses are frequently handled in the same area, and carcass preparation is carried out at the ambient temperature. After complete bleeding, the animals were skinned on the floor, eviscerated, shackled from hind legs, lifted manually to be hanged on a chain dropped from the abattoir ceiling, split into two sides, washed with water, and postmortem examined.

Just before the official stamping by authorized person, to be passed as fit for human consumption, the outer surfaces of the shoulder and thigh regions of 100 carcasses, during four visits (25 carcasses/visit), were sprayed for 30 seconds, with a solution of either 2% LA, 2% AA, 12% TSP (solution temperature was ~20°C), or distilled water (as a negative control), using a clean hand-pump sprayer. The nozzle angle of the sprayer was 50° flat spray, at pressure of 40 psi, and droplet size of 900 *μ*m. The spray nozzle oscillation speed was 60 cycles per min, and the distance from sample was 15 cm. Each treatment was applied onto 25 carcasses. The chemical decontaminants were bought from El-Nasr Pharmaceutical Chemicals Company (Cairo, Egypt) and were diluted with sterile distilled water to the appropriate concentration.

### 2.2. Sampling Techniques

Tissue excision samples were taken from the outer surface of shoulder and thigh of beef carcasses that were subjected to decontaminant substances, as well as from control matrices (50 tissue excision samples from each treatment, represented by 25 samples from each of the shoulder and thigh). The same sets of control carcasses sampled by excision were also sampled using surface swabbing (50 samples, represented by 25 surface swabbing samples from each of the shoulder and thigh). The surface swabbing was conducted only the first visit, as preliminary approach in order to define the better strategy to determine the microbial counts on beef carcass surfaces under the influence of the chemical decontaminants applied in the present study during the visits 2–4.

Surface excision technique was conducted by using a sterile coring punch to delimit an area of 10 cm^2^ and to cut the tissue to a depth of approximately 5 mm. The tissue was then excised using sterile scalpel and forceps and placed in a separate sterile stomacher bag, containing 100 ml of 0.1% sterile peptone water. For carcass surface swabbing, a sterile metal template, with an area of 10 cm^2^, was placed firmly against the examined surface of beef carcasses on an adjacent area of the tissue excision part. The limited area was swabbed with a first sterile cotton wool swab that was previously moistened in a 0.1% sterile peptone water, and then with a second dry sterile cotton wool swab. Both moistened and dry swabs were suspended in 10 ml sterile peptone water (0.1%). The collected samples in sterile stomacher bags were labeled, placed in isothermal containers, and transported to the laboratory, as quickly as possible, for microbiological analysis, in the same day.

### 2.3. Sample Preparation

Sample was prepared according to ISO 17604 [18]. As a first step, excised tissue samples were homogenized in a Stomacher 400 Lab Blender (60 seconds) (Seward Medical, London, UK). Next, the mixture was allowed to stand 15 minutes at room temperature. Subsequently, decimal serial dilutions were made, in the same sterile peptone water, in order to be used for microbiological analyses. For swabbing sample preparation, 1 ml from the original suspension was transferred aseptically, with sterile pipette, into a sterile test tube containing 9 ml of sterile peptone water (0.1%), resulting in a dilution of 10^−1^, from which further ten-fold decimal dilutions were prepared for microbiological analyses.

### 2.4. Microbiological Analyses

#### 2.4.1. Aerobic Plate Count

Aerobic plate counts (APC) were determined by inoculating 1 ml from the sample homogenate, at selected dilutions, onto duplicate sterile plates and thoroughly mixed with about 15 ml of previously adjusted (45 ± 1°C) standard plate count agar (CM325; Oxoid, Thermo Fisher Scientific Inc., UK). After solidification, the inoculated plates, as well as control one, were inverted and incubated at 35°C for 48 hours. Plates containing 30 to 300 colonies were counted, and the total colony count per cm^2^ was calculated and recorded [19].

#### 2.4.2. Enterobacteriaceae Count

*Enterobacteriaceae* counts (EBC) were obtained using the pour plating method on Violet Red Bile Glucose Agar (VRBGA; CM 485; Oxoid, UK). After solidification, the plates were overlaid with another 5 ml of the medium to suppress surface colonies. The inoculated plates, in addition to the control one, were incubated at 35°C for 24 hours [20]. All typical colonies characterized by purple color, higher than 0.5 mm in diameter, and surrounded by a zone of precipitated bile were counted and recorded as a total *Enterobacteriaceae* count/cm^2^. Biochemical identification of the isolated Gram-negative rods of *Enterobacteriaceae* family to genera, and, finally, to species level, were based on the performing of the following biochemical tests: oxidase test, motility test, urease production, hydrogen sulphide production, utilization of citrate, indole production, methyl red test, Voges-Proskauer test, sugar fermentation, and Eijkman test [21].

#### 2.4.3. Total Enterococci Count

Enterococcus selective differential medium (ESD; 45183-500G Merck, Germany) was used for the determination of the enterococci count. 0.1 ml of the diluted samples was spread evenly on duplicate ESD agar plates. Inoculated plates were incubated at 37°C for 48 hours, and the average count per cm^2^ was recorded.

#### 2.4.4. Most Probable Number of Coliforms

The recommended three-tube most probable number (MPN) method was adapted [22]. One milliliter of decimal dilution was inoculated separately into each of the three MacConkey broth tubes (CM5; Oxoid, UK) with inverted Durham's tubes were incubated at 37°C, then examined after 24 hours and 48 hours, respectively. Positive tubes showing acid and gas productions in inverted Durham's tubes were recorded. The most probable number of coliforms/cm^2^ was calculated [23].

#### 2.4.5. *Staphylococcus aureus* Counts

*Staphylococcus aureus* counts were determined on Baird-Parker agar medium (CM275; Oxoid, UK) with egg yolk–tellurite emulsion (SR54; Oxoid, UK). Plates were incubated at 37°C for 24–48 h. All typical colonies (lipase-positive jet-black colonies) on Baird-Parker agar were counted. Selected colonies from the agar surfaces were Gram stained, tested for catalase reaction, DNA-ase activity, and, subsequently, confirmed for exhibiting coagulase activity [21].

### 2.5. Statistical Analysis

The mean microbial counts of the examined samples were converted into base-10 logarithms of colony forming units per cm^2^ of the carcass surface (log_10_ CFU/cm^2^). One-way analysis of variance (ANOVA) for 4 independent samples was applied to determine the differences in microbial counts among the different treatments. Significant differences among the means were determined by Tukey honestly significant difference (HSD) test. One tailed *t*-test was conducted to identify the significance of the difference in mean counts between swabbing and tissue excision methods. The significance was defined at *P* < 0.05. All data analysis was performed using the VassarStats web site for statistical computation (http://faculty.vassar.edu/lowry/VassarStats.html).

## 3. Results and Discussion

In order to examine the efficiency of the tested LA, AA, and TSP, as organic decontaminating agents, we investigated their influence on the microbiological population of aerobic bacteria, *Enterobacteriaceae*, enterococci, coliforms, and *S. aureus*, respectively, present on the beef carcasses slaughtered in traditional abattoir in Zagazig, Egypt.

### 3.1. Swabbing versus Tissue Excision

**Figure 1:**
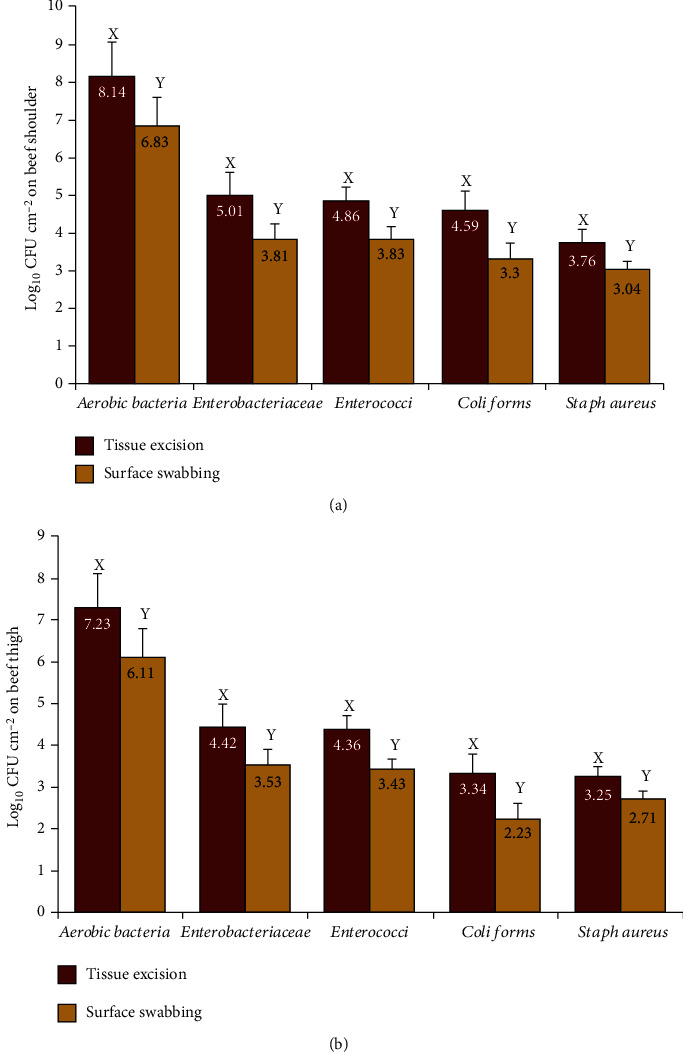
(a) Comparison between surface swabbing and tissue excision techniques for the recovery of aerobic count, *Enterobacteriaceae*, Enterococci, coliforms, and *Staphylococcus aureus* from the outer surfaces of shoulder of control beef carcasses (*n* = 25). Values represent means ± SE of the log counts. Columns with different letters within the same microbial category differ significantly (*P* < 0.05). (b) Comparison between surface swabbing and tissue excision techniques for the recovery of aerobic count, *Enterobacteriaceae*, Enterococci, coliforms, and *Staphylococcus aureus* from the outer surfaces of thigh of control beef carcasses (*n* = 25). Values represent means ± SE of the log counts. Columns with different letters within the same microbial category differ significantly (*P* < 0.05).

Both tissue excision and surface swabbing samples taken from the shoulder and thigh regions of the investigated carcasses were bacteriologically examined. The obtained results highlighted that the surface tissue excision method yielded significantly higher microbial counts (*P* < 0.05) for all of the five investigated bacterial categories than those obtained by swabbing method (Figures 1(a) and 1(b)). In this regard, the recovered APC, EBC, enterococci count, coliform, and *S. aureus* counts from the shoulder and thigh by excision technique were higher by nearly 1 log CFU/g than the corresponding counts obtained by swabbing method (Figures 1(a) and 1(b)).

The most reliable bacteria count by surface tissue excision method may be due to the complete recovery of firmly attached bacteria to meat surface [24]. Our results are in accordance with those reported by Gill et al. [25] and Hutchison et al. [26] who concluded that the obtained bacterial counts sampled by excision were significantly higher than measured by swabbing. However, other studies suggested that swabbing can be generally recovered comparable bacterial numbers with those obtained by excision [27, 28].

Considering that the sampling of meat surfaces by tissue excision method recovered more bacteria than sampling by swabbing, we used the excision technique to determine the microbial counts on beef carcass surfaces under the influence of the chemical decontaminants (LA, AA, and TSP) applied in the present study.

### 3.2. Aerobic Plate Count (APC)

**Figure 2:**
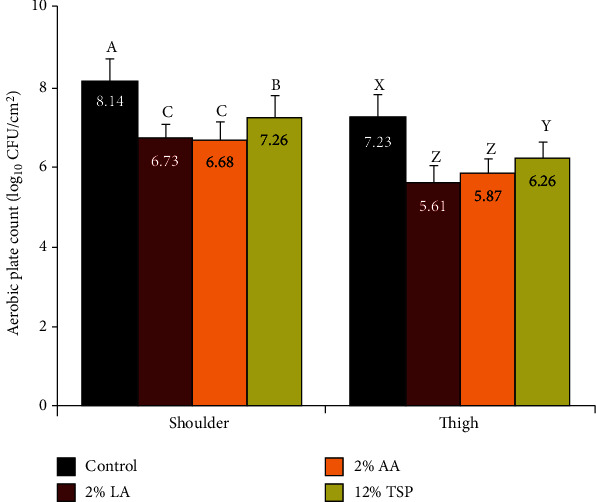
Effect of lactic acid (LA), acetic acid (AA), and trisodium phosphate (TSP) spraying for 30 s on the aerobic plate count (APC), as counted by tissue excision technique from the outer surfaces of shoulder and thigh of beef carcasses (*n* = 25). Values represent means ± SE of the log count of three replicates. Columns with different letters indicate significant differences (*P* < 0.05) between the sampling location of the slaughtered beef carcasses.

The registered mean of APC (log_10_ CFU/cm^2^) from shoulder samples obtained by excision method were 8.14, 6.73, 6.68, and 7.26 for control and 2% LA-, 2% AA-, and 12% TSP-treated samples, respectively, while the corresponding counts from the thigh samples were 7.23, 5.61, 5.87, and 6.26, respectively (Figure 2). It is generally recognized that bacterial counts which differ by <0.5 log unit are not substantially different [29]. Thus, the antimicrobial effects of decontaminating treatments must be regarded as inefficient, when the numbers of bacteria recovered before and after a treatment do not differ by at least 0.5 log unit. All of the treated samples in the present study exhibited significantly lower values of APC (*P* < 0.01), when they compared with the control samples. The LA, AA, and TSP treatments reduced APC in shoulder samples by 1.41, 1.46, and 0.88 logs, respectively, in comparison with the control samples, while a reduction counts of 1.62, 1.38, and 0.97 logs were encountered for LA-, AA-, and TSP-sprayed thigh samples, respectively, in comparison with the control group (Figure 2).

It was noticed that the registered APC on the surface of shoulder region, counted by excision method, was slightly higher than that observed by thigh region (8.14 vs. 7.23). This finding may be related to the fact that the shoulder area is near the floor of the abattoir, as the carcasses are hanged from the hindquarter and thus are more liable to a higher contamination. This result is in agreement with the findings reported by Yashoda et al. [30].

In accordance with our results, significant reductions in APC on beef carcasses had been reported for lactic acid spray [31, 32], acetic acid spray [33], and trisodium phosphate spray [34].

Our results indicate no significant difference (*P* > 0.05) in the reduction level of APC between LA and AA treatments, whilst the reduction effect of TSP was considerably less (*P* < 0.01) in comparison with that produced by AA- and LA-sprayed samples.

### 3.3. Enterobacteriaceae Count (EBC)

**Figure 3:**
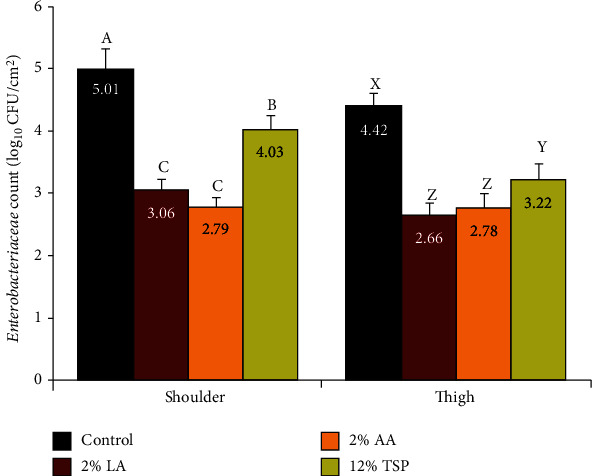
Effect of lactic acid (LA), acetic acid (AA), and trisodium phosphate (TSP) spraying for 30 s on the *Enterobacteriaceae* count, as counted by tissue excision technique from the outer surfaces of shoulder and thigh of beef carcasses (*n* = 25). Values represent means ± SE of the log count of three replicates. Columns with different letters indicate significant differences (*P* < 0.05) between the sampling location of the slaughtered beef carcasses.

The mean EBC (log_10_ CFU/cm^2^) obtained by excision method from shoulder samples were 5.01, 3.06, 2.79, and 4.03 for the control group and 2% LA-, 2% AA-, and 12% TSP-sprayed samples, respectively. The corresponding counts for thigh samples were 4.42, 2.66, 2.78, and 3.22, respectively (Figure 3). The high count of *Enterobacteriaceae* reported herein in control samples in comparison with the acceptable limit of 2.5 log_10_ CFU/cm^2^ by tissue excision method for *Enterobacteriaceae* count [35] may be attributed to the possible contamination of carcass surface with content from the gastrointestinal tract during dressing faults, evisceration, or handling.

All of the treatments showed significant (*P* < 0.01) reduction in EBC in comparison with the control group. In shoulder samples, AA treatment significantly (*P* < 0.01) reduced the EBC by 2.22 logs, while reduction counts of 1.95 and 1.01 logs in EBC were achieved by LA and TSP spraying, respectively, in comparison with the control. In thigh samples, however, LA, AA, and TSP spraying resulted in reduction of EBC by 1.76, 1.64, and 1.20 logs, respectively, when compared with controls.

Interestingly, AA and LA treatments reduced the EBC by higher log values than their reduction achieved in APC. The destructive action of lactic and acetic acids on proteolytic bacteria, particularly Gram-negative, is believed to be through induction of low pH and liberation of undissociated acid molecules that change the permeability microbial cell membrane [36].

EBC reduction under the influence of acetic and lactic acid [9, 37], as well as trisodium phosphate treatment [9, 34, 38] on meat surfaces, has been also reported in other studies.

Among the members of the *Enterobacteriaceae* family, there are many species which have been reported to cause health hazards to the consumers [39]. In addition, other species are considered important from economic point of view, as they are able to cause spoilage and deterioration of the raw meat [40]. Biochemical characterization of the recorded *Enterobacteriaceae* colonies at the species level demonstrated that *Citrobacter freundii*, *Enterobacter aerogenes*, *Enterobacter cloacae*, *Enterobacter sakazakii*, *Erwinia ananas*, *Escherichia coli*, *Hafnia alvei*, *Klebsiella planticola*, *Kluyvera ascorbata*, *Morganella morganii*, *Proteus vulgaris*, *Providencia rettgeri*, and *Serratia odorifera* could be isolated from the examined beef surfaces, with varying percentages ranging from 4% to 27%. The most prevalent *Enterobacteriaceae* isolated from shoulder surface were *Enterobacter* spp. (27%), followed by *Escherichia coli* (22%); meanwhile, the most prevalent isolated *Enterobacteriaceae* from thigh surface were *Citrobacter* spp. (25%), *Escherichia coli* (18%), and *Serratia odorifera* biogroup 1 (14%). However, in the present survey, the total counts of Enterobacteriaceae not reflect the presence of microorganisms with major public health concern (e.g., *Salmonella* or STEC serotypes). In this regard, further studies are still required to address this current challenge in condition of the surveyed area.

### 3.4. Total Enterococci Count

Enterococci are considered common bacteria of the intestinal tracts of animals. They are resistant to many unfavorable conditions and may contaminate the carcass surface during evisceration and handling. Thus, their role as index organisms for fecal pollution has been recognized.

**Figure 4:**
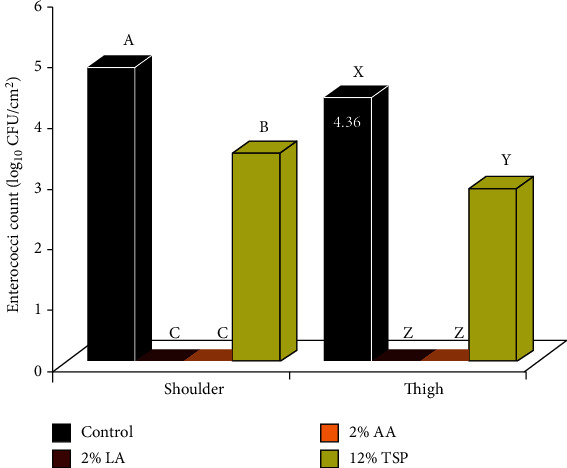
Effect of lactic acid (LA), acetic acid (AA), and trisodium phosphate (TSP) spraying for 30 s on the enterococci count, as counted by tissue excision technique from the outer surfaces of shoulder and thigh of beef carcasses (*n* = 25). Values represent means ± SE of the log count of three replicates. Columns with different letters indicate significant differences (*P* < 0.05) between the sampling location of the slaughtered beef carcasses.

The registered mean enterococci count (log_10_ CFU/cm^2^) in the control samples from shoulder and thigh regions obtained by tissue excision technique were 4.86 and 4.36, respectively (Figure 4). The application of spraying solution containing either 2% LA or 2% AA for 30 s on the outer surfaces of the shoulder and thigh regions resulted in a complete decline (<10 CFU) of the enterococci count (Figure 4), which indicated that LA and AA can be considered as very effective decontaminants against enterococci. This efficient decontaminant effect against enterococci may be attributed to the fact that the sprayed acid (2%) reduces the pH of meat surface to 2.6 [34], which is not suitable for the growth of enterococci, which prefer alkaline media (proper pH for enterococci growth is 9.6).

On the other hand, spraying of beef carcass surfaces with 12% TSP significantly (*P* < 0.01) reduced the enterococci count, by 1.43 and 1.51 logs from the outer surface of shoulder and thigh, respectively, in comparison with the control group.

Enterococcus species play a role as an opportunistic pathogen in extraintestinal diseases, such as endocarditis, urinary tract, intra-abdominal, central nervous, and pelvic infections in humans [10, 41]. They have a high level of heat resistance and often survive in marginally processed meat and meat products. Enterococci can be associated with food-borne infections, because of their frequent presence in foods and are capable of spreading virulence or resistance genes throughout the food chain [42]. Additionally, enterococci can cause food intoxication through production of biogenic amines and can be a reservoir for troublesome opportunistic infections and for virulence traits [10].

### 3.5. Most Probable Number (MPN) of Coliforms

Enteric organisms such as coliforms frequently contaminate meat, indicating that the gut is a common source of contamination. Presence of coliforms in meat is a useful and reliable indicator of its contamination, being an indicator for faulty method of slaughtering, carcasses preparation, and handling [21].

**Figure 5:**
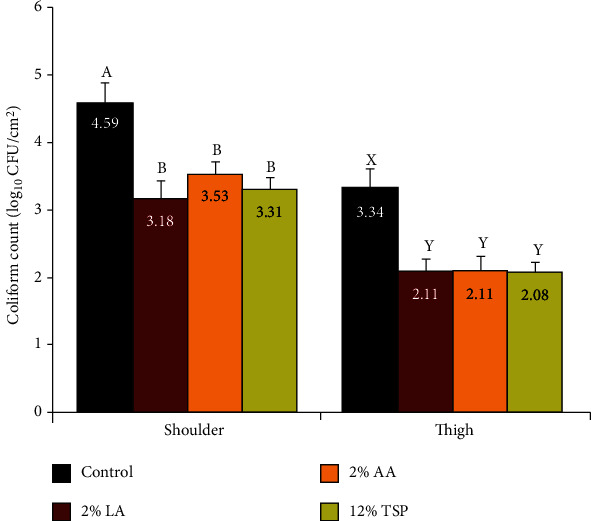
Effect of lactic acid (LA), acetic acid (AA), and trisodium phosphate (TSP) spraying for 30 s on the most probable number (MPN) of coliforms, as counted by tissue excision technique from the outer surfaces of shoulder and thigh of beef carcasses (*n* = 25). Values represent means ± SE of the log count of three replicates. Columns with different letters indicate significant differences (*P* < 0.05) between the sampling location of the slaughtered beef carcasses.

The mean values of MPN of coliforms (log_10_ CFU/cm^2^) were 4.59 and 3.34 as counted from shoulder and thigh surfaces, respectively (Figure 5). The significantly higher coliform count particularly in control shoulder samples, comparing with the maximal permissible limit of 3.5 log_10_ CFU/cm^2^ [43], may be related to the existing poor sanitary conditions at the abattoir.

Significant (*P* < 0.01) reduction in the MPN of coliforms is attained under the influence of LA, AA, or TSP treatments. Spraying of the outer surface of beef shoulder with 2% LA, 2% AA, and 12% TSP for 30 s reduced MPN of coliforms by 1.41, 1.06, and 1.28 logs, respectively, as counted by tissue excision method. The corresponding reductions in MPN of coliforms achieved by such treatments in thigh samples were 1.23, 1.23, and 1.26 logs, respectively (Figure 5).

Coliform counts reflect inadequate sanitation during production and handling of raw material, meat contact surfaces, and employees. Meanwhile, the occurrence of large numbers of them on carcass surfaces is highly undesirable and suggests mostly fecal contamination and points to potentially severe hazard for the consumer [44].

### 3.6. Staphylococcus Aureus

Considering that staphylococci are commonly found on the skin of a wide variety of mammals and birds and on environmental surfaces, humans and, in particular, food handlers are thought to be the primary source of strains associated with food origin *Staphylococcus aureus* intoxications [45].

**Figure 6:**
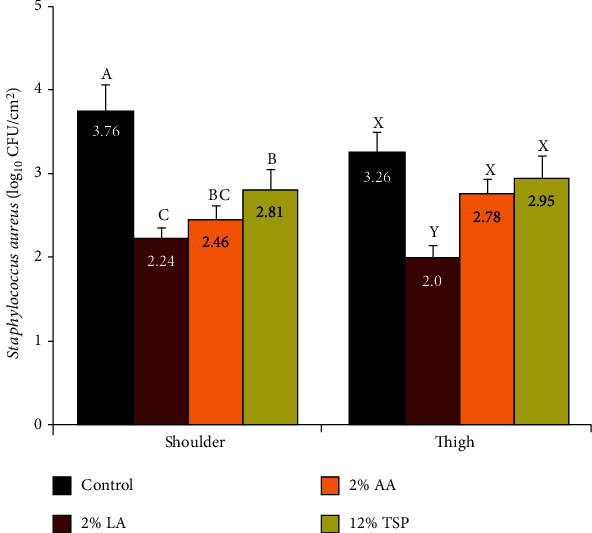
Effect of lactic acid (LA), acetic acid (AA), and trisodium phosphate (TSP) spraying for 30 s on the *Staphylococcus aureus* count, as counted by tissue excision technique from the outer surfaces of shoulder and thigh of beef carcasses (*n* = 25). Values represent means ± SE of the log count of three replicates. Columns with different letters indicate significant differences (*P* < 0.05) between the sampling location of the slaughtered beef carcasses.

The mean count of *S. aureus* from shoulder samples was 3.76 log_10_ CFU/cm^2^, as counted by surface excision method. Spraying of LA, AA, and TSP on the outer surface of the examined shoulder for 30 s significantly reduced (*P* < 0.01) the *S. aureus* counts by 1.52, 1.30, and 0.95 logs, respectively, (Figure 6), when compared with the controls.

In thigh samples, the mean *S. aureus* count was 3.26 log_10_ CFU/cm^2^, as counted by surface excision method. Spraying of the outer surface of the examined thigh with 2% LA significantly (*P* < 0.01) reduced the *S. aureus* counts with 1.26 logs. Contrary, AA and TSP spraying did not induce significant reduction to the *S*. *aureus* count in thigh samples, although they exhibited 0.48 and 0.31 logs, respectively, lower than the controls (Figure 6).

These results highlighted that LA was the most effective decontaminant against *S*. *aureus*, followed by AA, while TSP produced the weakest effect. In this context Dubal et al. [11] observed that the inoculated *S*. *aureus* onto sheep and goat forequarters was completely inhibited by spraying of 2% lactic acid for 2-4 min.

It was noticed that the reduction values by TSP were considerably lesser (*P* < 0.05) than those induced by LA or AA. These findings have been previously reported by Rhône-Poulenc [46], which observed that the TSP killing procedures are aimed primarily at Gram-negative pathogenic and spoilage bacteria and are relatively ineffective against Gram-positive spoilage bacteria.

Application of some organic acids comprising lactic and acetic acids for decontaminating carcass surfaces causes either cell death or sublethal cellular injury of bacterial cells [9]. The antimicrobial activity of LA and AA is attributed to both the metabolic inhibition by the undissociated LA and AA molecules and to the depression of pH below the growth range of many bacteria [47].

Considering the poorly applied hygienic standards in the investigated abattoir and in such others in Egypt, the application of carcass decontamination practices can represent a useful tool in the improvement of the meat microbiological quality. However, it is important to note that there is a criticism trend on the decontamination based on two major reasons, namely, (i) the remediation of the improper meat quality does not stimulate the adoption of better slaughtering practices and (ii) the meat decontamination might unbalance the competition between the spoilage microbiota and pathogens, which can lead to the fact that the decontaminated meat might become more hazardous than that of the noncontaminated.

## 4. Conclusion

The study results highlighted that the application of LA and AA, as well as TSP can greatly reduce the microbial population on the meat surfaces of the beef carcasses, and hence they can be applied as microbial decontaminants on beef carcass surfaces which subjected to high degree of contamination in the traditional abattoir in Egypt. Furthermore, our results indicated that the antimicrobial effects of the used decontaminants followed the order LA>AA>TSP.

## Data Availability

The data used to support the findings of this study are included within the article.
